# Root Digger: a root placement program for phylogenetic trees

**DOI:** 10.1186/s12859-021-03956-5

**Published:** 2021-05-01

**Authors:** Ben Bettisworth, Alexandros Stamatakis

**Affiliations:** 1 Computational Molecular Evolution Group, Heidelberg Institute for Theoretical Studies, Heidelberg, Germany; 2 Institut für Theoretische Informatik, Karlsruhe Institute of Technology, Karslruhe, Germany

**Keywords:** Phylogenetic analysis, Phylogenetic rooting, Maximum likelihood

## Abstract

**Background:**

In phylogenetic analysis, it is common to infer unrooted trees. However, knowing the root location is desirable for downstream analyses and interpretation. There exist several methods to recover a root, such as molecular clock analysis (including midpoint rooting) or rooting the tree using an outgroup. Non-reversible Markov models can also be used to compute the likelihood of a potential root position.

**Results:**

We present a software called RootDigger which uses a non-reversible Markov model to compute the most likely root location on a given tree and to infer a confidence value for each possible root placement. We find that RootDigger is successful at finding roots when compared to similar tools such as IQ-TREE and MAD, and will occasionally outperform them. Additionally, we find that the exhaustive mode of RootDigger is useful in quantifying and explaining uncertainty in rooting positions.

**Conclusions:**

RootDigger can be used on an existing phylogeny to find a root, or to asses the uncertainty of the root placement. RootDigger is available under the MIT licence at https://www.github.com/computations/root_digger.

## Background

In standard phylogenetic inference, most tools [1, 2] yield unrooted trees. This is because they typically implement time-reversible nucleotide substitution models [3] as they yield the phylogenetic inference problem computationally tractable. However, time-reversible models are incapable of identifying the root, as they disregard the direction of evolution. This is the result of the so-called pulley-principle [3]. Nevertheless, a rooted phylogeny is often required for downstream analyses and interpretation of results as it can resolve long standing disputes regarding the placement of large clades on the tree of life for example [4]. Often then, researchers will have to use a dedicated tool or include additional information in the analysis to recover the root of an inferred *unrooted* phylogenetic tree.

To root a tree when the primary phylogenetic inference is performed via a reversible model, researchers typically deploy one of the two following methods: including a set of outgroup taxa in the analysis, or using some form of molecular clock analysis. Unfortunately, both approaches exhibit their own challenges and pitfalls [5]. Including outgroup taxa in the analysis increases the amount of work that must be conducted in order to infer a tree, and, more importantly, can also affect the ingroup topology in unexpected ways [6]. Molecular clock analysis can be complicated by the need to calibrate the molecular clock, as this often requires appropriate and a sufficient number of fossil records that are related to the organisms under study [5, 7].

Alternatively, one can use a non-reversible model as, under such a model, the root placement *does* affect the likelihood of the tree [8]. Examples of non-reversible models include gene tree species tree reconciliation methods that account for gene duplication, loss, and transfer [9], or non-reversible Markov substitution processes for character evolution. It is the latter process that RootDigger uses to root an existing phylogeny. This allows RootDigger to circumvent the compute-intensive step of inferring a tree under a non-reversible model, and instead only use a non-reversible model to root the inferred tree in a final step. By doing this, one can combine the advantages of both, fast tree inference under reversible models, and rooting the tree under a non-reversible model.

The rest of this paper is organized as follows. First, we provide some more background on the theory and operation of RootDigger, as well as a justification for our method. Then, we describe the operation of RootDigger in detail. Next, we outline the methodology used to experimentally verify RootDigger and present the respective results. Finally, we discuss the effectiveness of RootDigger.

Methods that use additional topological information take advantage of prior knowledge about the world, which is not present in the, generally molecular, data that is used to infer the tree. In particular, knowledge about specific species which are not too distantly related to the species in question can be included as a so-called outgroup. This outgroup can then be used to place the root on the tree, as the most recent common ancestor of the ingroup and the outgroup should be the root of the tree.

There are challenges to including an outgroup to an analysis. Gatsey et al.  [10] showed how adding a single taxon to an analysis can substantially change the resulting tree topology, even for the taxa which were already present in the analysis (i.e., the ingroup). Holland [6] investigates this phenomenon in simulations, and finds that outgroups that affect or alter the ingroup topology are common.

Alternatively, molecular clock analysis can be used to place a root without prior topological knowledge [11]. The molecular clock hypothesis assumes that the substitution process exchanges bases (i.e., “ticks”) at a stochastically constant rate. Using this supposition a likely location can be inferred for the root on an existing phylogenetic tree. A simple version of this is midpoint rooting, which relies on a constant molecular clock assumption in order to produce a phylogenetically meaningful rooting yet it can be applied to any binary tree, regardless of whether it is ultrametric or not. Other methods, such as Minimum Ancestor Deviation (MAD) [12] and MinVar [13] also rely on the molecular clock assumption. They attempt to solve the potentially poor performance of midpoint rooting in the presence of a violation of a strict molecular clock by allowing for variation in the rate of the molecular clock.

Molecular clock analyses exhibit their own difficulties. In particular, the clock does not generally “tick” at a constant rate over the tree [14, 15]. Relaxed clock models exist which can alleviate this problem, but are not always successful at correctly identifying the root as shown in [5] and come with their own set of inference errors and methodological challenges.

The final method that can place a root on a tree is to perform the phylogenetic analysis under a non-reversible model of evolution. When using a non-reversible model, the direction of time affects the likelihood of the tree [8]. Using this property, the most likely location on the tree for the root can be found, so long as the model has an appropriate fit. Indeed, early results suggested that some non-reversible models (particularly those based on character substitution) are inappropriate for the purposes of rooting a tree [16]. However, in this work we find that these concerns appear to be mostly overstated (see Results). Several software packages are able to infer or score a phylogenetic tree under a non-reversible model, and as a by-product also identify a root [1, 17].

Non-reversible models for phylogenetic trees come in many forms. For example, accounting for duplication, transfer, and loss events yields a non-reversible model [9]. In particular, duplication events have been used for rooting trees [18]. Another method, the one primarily used in this work, is to eliminate the reversibility assumption of standard character (e.g., nucleotide or amino acid) substitution models. Unfortunately, eliminating this assumption significantly increases the computational effort required to find a good (high likelihood) phylogenetic tree. This is due to the resulting inapplicability of the pulley principle [3], which allows phylogenetic inference tools to ignore root placement during tree inference. Therefore, by adopting a non-reversible model, the location of the root on a phylogenetic tree affects the likelihood of that tree.

As the location of the root affects the likelihood of the tree, when using standard tree search techniques all possible rootings would need to be evaluated for each tree considered in order to find the rooting with the highest likelihood. In the worst case, this increases the work *per tree* being visited during the tree search by a factor of ${\mathcal {O}}(n)$ where *n* is the number of taxa in the dataset. Therefore, eliminating the reversibility assumption drastically increases the computational effort required to infer a tree. Hence, standard inference tools choose to adopt the reversibility assumption, as phylogenetic tree inference would be computationally intractable otherwise.

As an alternative to the computationally expensive process of inferring a tree with a root, an unrooted tree which has already been inferred under a reversible model can be evaluated a posteriori for possible root locations under a non-reversible model. This requires less computational effort, as it skips the expensive step of looking for “good” rootings in intermediate trees during the tree search. With this method, we can find the most likely root location for a given phylogenetic tree. Even this approach still faces numerical challenges, as previous research suggests that the likelihood function for rooting a phylogenetic tree may exhibit several local maxima [16], although we did not find this to be a major issue in our experiments (see discussion).

We implemented the open source software tool RootDigger which uses a non-reversible model of character substitution to infer a root on an already inferred, given tree. The inputs to our tool are a multiple sequence alignment (MSA) and an unrooted phylogenetic tree. RootDigger then returns a rooted tree. RootDigger implements fast and a slow root finding modes, called Search mode and Exhaustive mode, respectively. The search mode simply finds the most likely root quickly via appropriate heuristics, and is intended for users who simply intend to root the tree. For a more through exploration of the possible roots, we provide the exhaustive mode, which thoroughly evaluates the likelihood of placing the root into every branch of the given tree, and reports the likelihood weight ratio [19] for placing a root on that branch for every branch on the tree. In other words, the exhaustive mode allows to quantify root placement uncertainty.

Additionally, RootDigger supports both thread and process level parallelism, over the potential data partitions of phylogenomic alignments and over distinct search starting locations (i.e., parallelization of the root search procedure), respectively. Finally, to support root inferences on extremely large datasets using compute clusters, we have implemented a checkpoint system in RootDigger, which allows for the search to be halted and resumed at a later point in time in case of hardware failures or when the job time limit has been reached.

## The Software

Usage of RootDigger is straight-forward. All that is required is a tree in newick format, and a MSA in either PHYLIP or FASTA format. RootDigger is open source, released under the MIT license, and written in C++, and is targeted at the Linux platform. The code, documentation, test suite, as well as any modifications to existing libraries can be found at https://www.github.com/computations/root_digger.

In order to implement both, likelihood computations, and non-reversible models, RootDigger has three major dependencies: the GNU Scientific Library (GSL) [20], the Phylogenetic Likelihood Library (LibPLL) [21], and L-BFGS-B [22]. GSL is used for the decomposition of the non-symmetric substitution rate matrix, LibPLL is used for efficient likelihood calculations, and L-BFGS-B is used for multi-parameter optimizations.

## Implementation

The input to RootDigger is a MSA and a phylogenetic tree with branch lengths in expected mean substitutions per site. RootDigger then uses the tree and branch lengths to find the most likely root location by calculating the likelihood of a root location under a non-reversible model of DNA^1^ substitution (specifically, UNREST [23] with a user specified number of $\Gamma$ discrete rate categories, and an optional proportion of invariant sites, i.e., UNREST+$\Gamma$+I). The UNREST model is used because numerous other models (including models which are in the Lie group detailed in [24]) have been derived from this model. The optimal position of the root along a specific branch of length *t* is calculated by splitting the given branch in two with resulting branch lengths $\beta t$ and $(1-\beta ) t$, with $0 \le \beta \le 1.0$. We then find the maximum likelihood value of $\beta$, and report the likelihood for the given branch as the likelihood of the root location on that branch. By formulating the problem this way, we can use single parameter optimization techniques such as Brent’s Method, which are computationally more efficient compared to multi-parameter optimization routines such as the BFGS algorithm (named for its creators: Broyden, Fletcher, Goldfarb, and Shanno). Note that we specifically selected Brent’s Method instead of Newton’s Method, because it does not require the calculation of the second derivative to optimize the function. While an analytical computation of the second derivative could be implemented, initial estimates showed that the savings were not sufficient to justify the increased complexity and potential numerical issues. Nonetheless, in principle, the computation of the second derivative of the likelihood is feasible and could be implemented.

A potential problem of Brent’s and analogous methods is that they find extrema by identifying roots for the derivative of the objective function. In order to find maxima, though, it is required that the objective function’s value is evaluated, as a root of the derivative could correspond to a minimum. In addition, Brent’s method will fail to find all extrema. To alleviate this, we need to search for bracketing windows that can be used to safely find extrema. Unfortunately, we are not aware of a general method for finding such bracketing windows, so a recursive method is employed, were the search range is bisected and adequately searched for appropriate windows. Appropriate here means that the sign of the function in question has opposite signs at the respective endpoints of the window.

As already mentioned, RootDigger offers two modes of operation. These modes will be discussed individually, starting with search mode:

1. Initialize numerical model parameters:
  $\alpha$-shape parameter for discrete $\Gamma$ rates to 1.0 (if applicable),
  Character substitution rates to $\frac{1}{4(4-1)} = \frac{1}{12}$
  Base frequencies to $\frac{1}{4}$
2. According to one of the following strategies (default 1% of possible root positions)
  Modified MAD (Default) or,
  Randomly.
3. For each starting root:
  1. Optimize model parameters
    $\alpha$-shape parameter for $\Gamma$ distributed rates (if applicable, and only every 10 iterations),
    Character substitution rates,
    Base Frequencies.
  2. Find the best root location for the current model
    1. Create a list of high likelihood root locations evaluated at the midpoint of every branch.
    2. For the top roots (default 1%), optimize the root location along their specific branch.
  3. Repeat from 3(*a*) until a stopping condition is met:
    The difference between likelihoods between the current iteration and the previous iteration is sufficiently small (below user defined parameter atol),
    If early stopping is enabled, the new root location is sufficiently close to the old root location by distance along the branch (below user defined parameter brtol) or,
    More than 500 iterations have passed.
4. Report the best found root, along with its log-likelihood

In order to select the initial branches in search mode, we have developed two strategies: modified MAD and random selection. When using modified MAD, we compute the approximate MAD ranking for each branch via a simplified version of the MAD algorithm for the purposes of computational efficiency. This approximate metric is used to rank branches for selection as initial root positions. There is a possibility that this option will bias the results, so we also provide a random branch strategy for these cases.

During the search, we re-estimate the base frequencies in every iteration to sufficiently optimize the likelihood, and because the cost of optimizing these parameters is small (approximately 10% of overall run time). Furthermore, because we use a non-reversible substitution matrix, the base frequencies might not be stable across every branch of the tree. Therefore, to ensure a good fit, we need to optimize the base frequencies every time. The algorithm for the exhaustive mode is analogous; the core optimization routines are the same as in search mode. The major difference is that now, all branches are being considered:

1. For every branch on the tree:
  1. Place root at current branch.
  2. Initialize numerical parameters:
    $\alpha$-shape parameter for discrete $\Gamma$ rates to 1.0 (if applicable),
    Character substitution rates to $\frac{1}{4(4-1)} = \frac{1}{12}$
    Base frequencies to $\frac{1}{4}$
  3. Optimize model parameters
    $\alpha$-shape parameter for $\Gamma$ (if applicable, and only every 10 iterations),
    Character substitution rates,
    Base Frequencies.
  4. Repeat from 1(*c*) until a stopping condition is met:
    The difference between likelihoods between this iteration and the previous iteration is sufficiently small (below atol) or,
    If early stopping is enabled, the new root location is sufficiently close to the old root location by distance along the branch (below brtol).
    More than 500 iterations have passed.
2. Report the tree with annotations for every branch:
  The root position along the branch,
  The log-likelihood,
  and the Likelihood Weight Ratio [19].

We re-initialize the initial model parameters in every iteration (from (3) in search mode and (1) in exhaustive mode) to avoid the numerous local minima, as discussed in [16]. In both modes, there is an upper limit to the number of iterations of 500. In empirical and simulated datasets this limit has never been reached, and only exists to ensure that the program will eventually halt.

In addition to the two search modes, there is an optional early stop mode, which can be combined with either of the root search modes. In this early stop mode, the search will terminate if the root placement is nearly the same twice in a row. This is to say, if the location of the best root position is on the same branch as in the previous iteration *and* the value inferred for the root position on that branch is sufficiently close the position in the previous iteration, the program will terminate. While the early stop optimization does improve rooting times substantially (approximately by a factor of 1.7 on some empirical datasets), the likelihood of each root placement will not be fully optimized. In practice, this does not substantially affect the final root placement, but it does render comparison of the likelihood with results from other tools invalid.

We utilize both OpenMP [25] and MPI to parallelize parts of the computation. First, we use the thread level parallelism of OpenMP to optimize each partition (sections of the alignment which are given their own model parameters) independently. If there are too few partitions present in the dataset to achieve ’good’ parallel efficiency, we also parallelize the transition matrix calculations over the branches. We use process level parallelism to parallelize searches over the initial search locations. This is most efficient in exhaustive mode, where there are many independent searches that can be carried out in parallel. To synchronize the processes, the results from each independent search are written to an append only binary log file. By using an append only file, synchronization of file locations is handled by the underlying filesystem, simplifying multinode checkpointing. At the end of the search, the results (root locations and their associated log-likelihoods, as well as the associated model parameters) in the checkpoint filed are reviewed, and the final step of finding the best root is performed by the master node. Using this strategy, we are able to (with sufficient independent searches) achieve a ’good’ parallel efficiency of 0.58 (see Fig. 12) **on how many cores?**. Furthermore, by using this append only logging method, we can also implement check pointing for the search. If the computations are interrupted during the search, when the search is re-started, the previous results are taken into account, and the search continues where it left off. In order to ensure that no write corruption has occurred during and that all writes are complete, a checksum is computed. To compute the checksum, we use the Alder-32 algorithm, which is implemented as a part of zlib [26]. To avoid a dependency on zlib for the checksum RootDigger includes the algorithm in its own code base.

## Results

To validate RootDigger, we conducted several experiments on both simulated and empirical data. Furthermore, we also used Likelihood Weight Ratios (LWR) [19] to asses the confidence of root placements on empirical datasets. Finally, we investigated the effects of the early stop mode on the final results.

### Experimental design

In the following sections we will describe the experimental setup for both simulated data and empirical data. Here, we will describe how we measured and computed the error for each of the methods. For simulations and empirical data, we computed the *topological* distance from the estimated root (by both IQ-TREE and RootDigger in search mode) to the true root, and normalized it by the number of nodes in the tree (both internal nodes as well as tips). If the correct root is picked, the distance is zero. For empirical data, the true root was taken to be the root indicated by the outgroup.

Evaluating the exhaustive search mode is difficult, since to our knowledge there are no other tools which perform the same task. Instead, we show the LWR distributions of empirical data which have been annotated onto trees. Additionally, these trees have the true root (again, as indicated by the outgroup) indicated.

#### Simulations

Tests with simulated data were conducted to both, validate the software, and to compare against IQ-TREE version 2.0.4 [27] which also implements the non-reversible UNREST model. We created a pipeline to

1. Generate a random rooted tree with ETE3 [28] and random model parameters.
  Substitution parameters for INDELible were generated by drawing uniformly between 0.01 and 1.01.
  Frequency parameters for INDELible were generated by an exponential distribution and then normalizing the parameters so that the frequency parameters sum to 1.
  Otherwise, options for INDELible were left to defaults.
  Branch lengths were generated via an exponential distribution using a scale parameter of 0.5
2. Simulate an MSA with indelible [29]
3. Execute RootDigger and IQ-TREE [27] with the simulated MSA, given the generated random tree.
4. Repeat from (2) for a total of 100 iterations
5. Compute comparisons
  1. Calculated rooted RF distance with ETE3 [30]
  2. Mapped root placement onto original tree with the true root.

Both IQ-TREE and RootDigger were given the same model options for all runs. RootDigger was executed with the arguments.

$${\texttt {rd}} \, \texttt {-}{} \texttt {-}{\texttt {msa}} \, {<}{\texttt {MSA FILE}}{>} \, \texttt {-}{} \texttt {-}{\texttt {tree}}\, {<}{\texttt {TREE FILE}}{>}$$

By default RootDigger uses no $\Gamma$ rate categories, and currently only supports the UNREST model [23]. IQ-TREE was executed with the arguments

$${\texttt {iqtree2}} \, {\texttt {-m 12.12}}\, {\texttt {-s}} \, {<}{\texttt {MSA FILE}}{>}\, {\texttt {-te}} \, {<}{\texttt {TREE FILE}}{>}$$

**Fig. 1:**
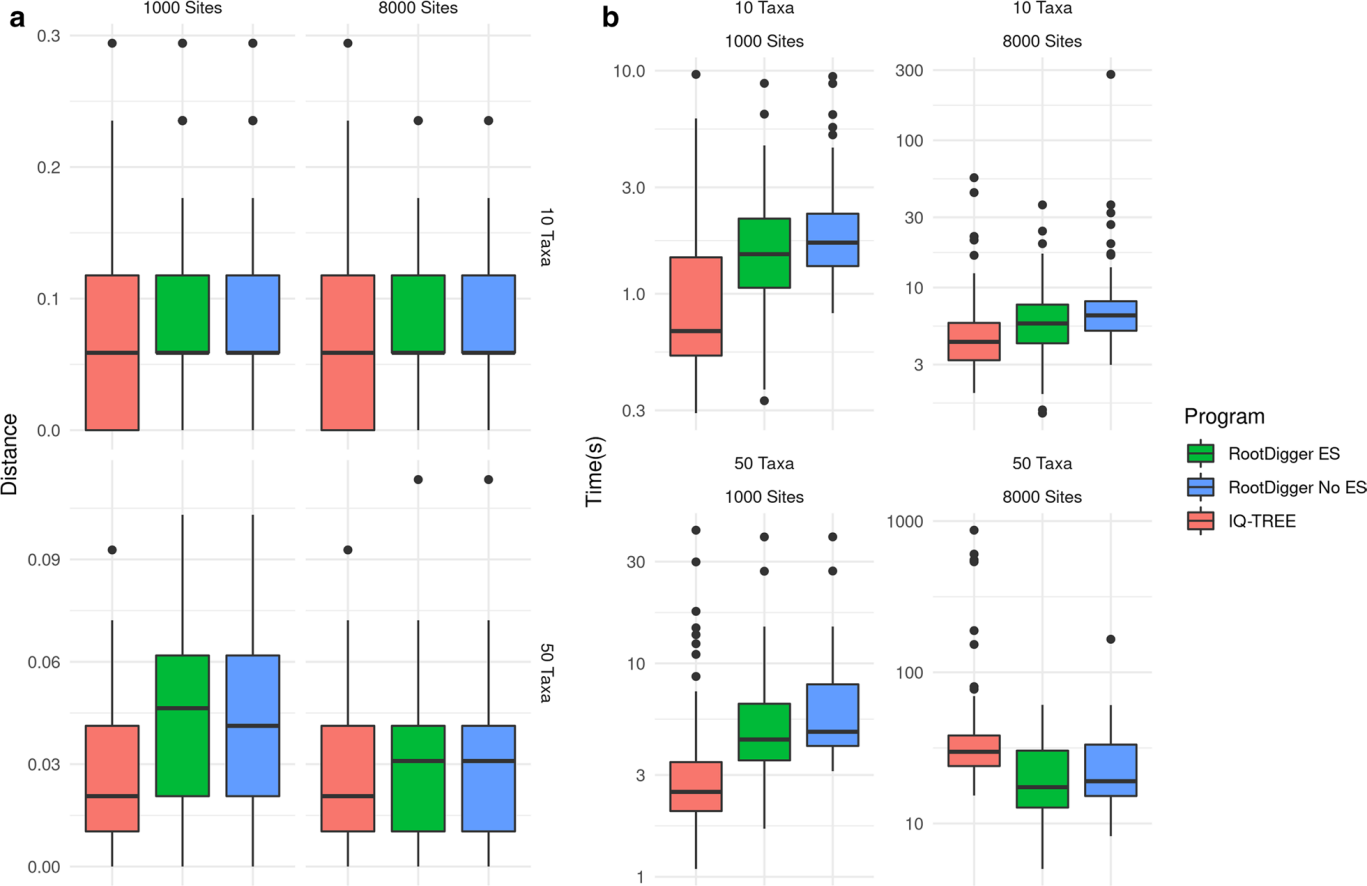
Box plot of results and execution times for IQ-TREE and RootDigger on simulated data with and without early stopping enabled

The -m 12.12 argument to IQ-TREE specifies that the UNREST model should be used [24] and the ${\texttt {-te}} \, {<}{\texttt {TREE FILE}}{>}$ option constrains the tree search to the given user tree. When given a fully resolved unrooted tree, this has the effect of rooting the tree. We used this option to simulate the operation of RootDigger. For all runs, the UNREST model was used. Furthermore, we vary two additional parameters to control dataset size: the number of MSA sites and taxa. In total, we ran 9 simulated trials with MSA sizes of 1000, 4000, and 8000 sites as well as tree sizes of 10, 50, and 100 taxa. The results from these experiments, as well as the execution times, are shown in Fig. 1.

#### Empirical Data

In addition to simulated data, we conducted tests with empirical data using IQ-TREE and additionally MAD [12]. The datasets used are described in Table 1 with additional statistics about the datasets in Table 2. The empirical datasets were chosen from TreeBASE [31, 32] and helpfully provided by fellow researchers [33] to include an existing, strongly supported outgroup. For each of the empirical datasets, we ran RootDigger in exhaustive mode to obtain likelihood weight ratios (LWR) for each branch. We ran the experiments on the datasets with the outgroup included, as well as with the outgroup excluded.

**Table 1:** Table of empirical datasets used for validation

Name	Dataset	Original model	Model used	Source
DS1	AngiospermsCDS12	GTR+$\Gamma$	UNREST + $\Gamma$4	[35]
DS2	AngiospermsCDS	GTR+$\Gamma$	UNREST + $\Gamma$4	[35]
DS3	Grasses	GTR+ G + I	UNREST + $\Gamma$4	[36]
DS4	Ficus	GTRCAT	UNREST + $\Gamma$4	[37]
DS5	SpidersMissingSpecies	NA^a^	UNREST + $\Gamma$4	[38]
DS6	SpidersMitocondrial	NA^a^	UNREST + $\Gamma$4	[38]
DS7	Beetles	GTR+G4	UNREST + $\Gamma$4	[33]
DS8	BeetlesHomogeneous	GTR+G4	UNREST + $\Gamma$4	[33]

^a^The paper states that PartitionFinder was used, but the results were not provided.

^b^The dataset is partitioned, and the partition file was provided. UNREST was used instead of any substitution matrices, but invariant sites and rate categories was preserved

**Table 2:** Table of statistics for the emperical datasets

Name	Tree diameter^a^	Root branch length^b^	Ratio^c^	#Genes	#Taxa	#Sites
DS1	1.1204	0.2276	0.203	1308	35	864,029
DS2	0.5236	0.1089	0.208	1308	35	1,296,043
DS3	0.6657	0.0856	0.129	3	245	4,973
DS4	0.0985	0.0316	0.320	5	200	5,552
DS5	0.0628	0.0099	0.158	1019	33	1,097,842
DS6	0.4189	0.0283	0.068	15	34	12,479
DS7	2.5334	0.0842	0.033	2948	14	4,098,894
DS8	1.6601	0.0539	0.032	101	14	186,499

^a^Defined here to be the longest path between two taxa.

^b^The length of the root branch if the tree was *unrooted*.

^c^Root branch length over tree diameter

We also performed some preprocessing. In order ensure that all branch lengths in all trees used were specified in substitutions per site, the branch lengths were re-optimized using RAxML-NG [34] version 0.9.0git. The original model was used when known, otherwise the branch lengths were optimized under GTR+$\Gamma$4.

Annotations are suppressed for branches with a small LWR (less than 0.0001). The trees with annotated LWR are shown in Figs. 2, 3, 4, 5, 6, 7, 8, 9, and 10. The analysis errors are summarized in Table 3 and runtimes for each method are summarized in Table 4.

**Fig. 2:**
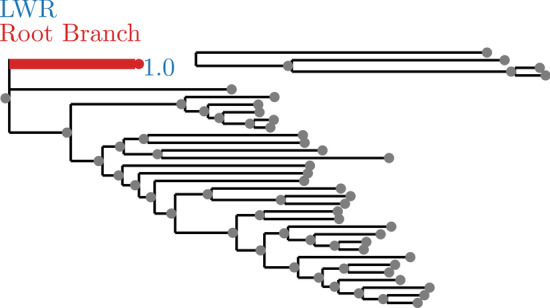
SpidersMissingSpecies dataset analyzed without an outgroup. LWR is the Likelihood weight ratio of placing a root on the branch. The true root branch is indicated in red

**Fig. 3:**
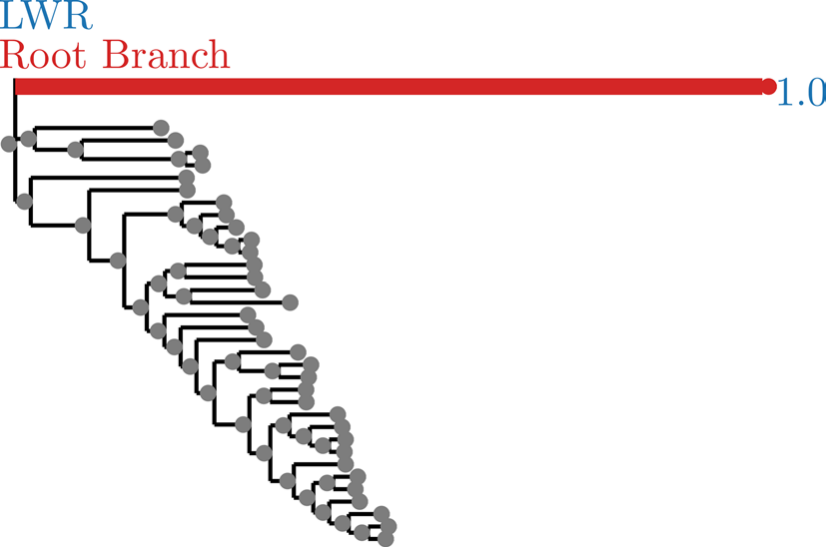
SpidersMissingSpecies dataset analyzed with an outgroup. LWR is the Likelihood weight ratio of placing a root on the branch. The true root branch is indicated in red

**Fig. 4:**
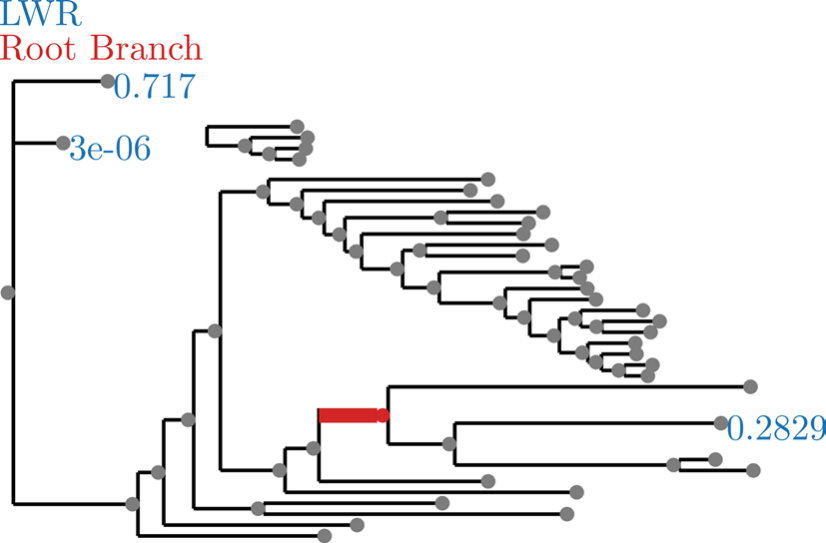
SpidersMitocondrial dataset analyzed without an outgroup. LWR is the Likelihood weight ratio of placing a root on the branch. The true root branch is indicated in red

**Fig. 5:**
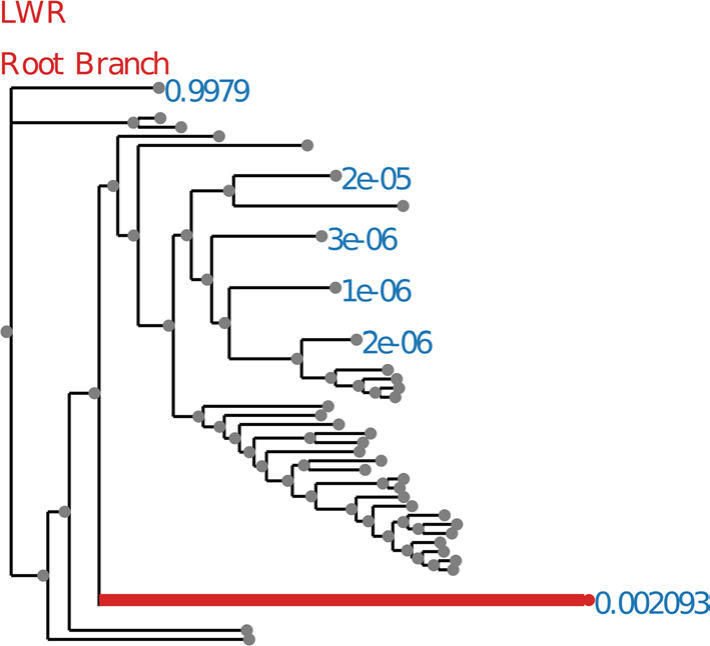
SpidersMitocondrial dataset analyzed with an outgroup. LWR is the Likelihood weight ratio of placing a root on the branch. The true root branch is indicated in red

**Fig. 6:**
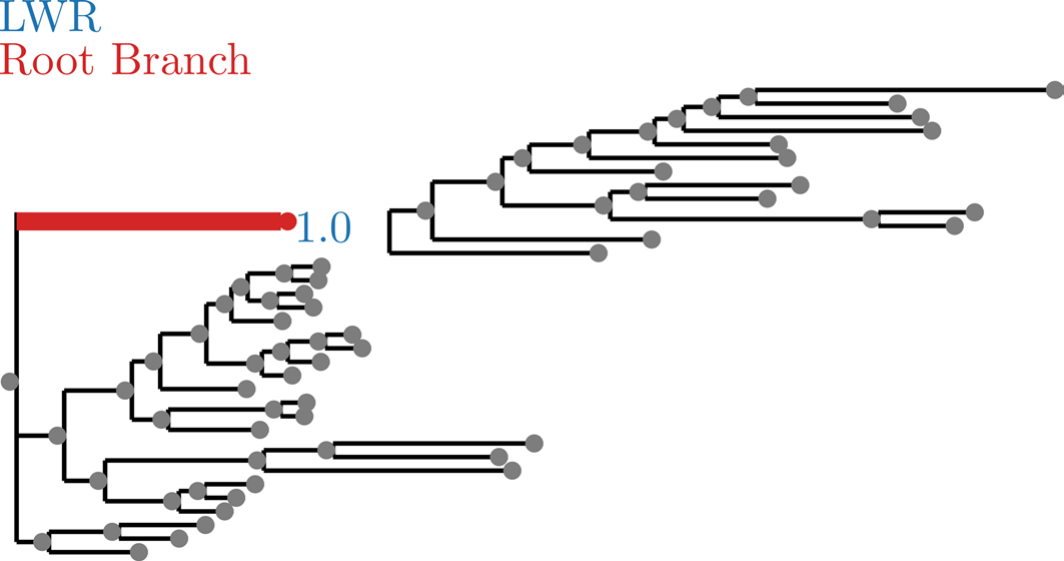
AngiospermsCDS12 dataset analyzed without an outgroup. LWR is the Likelihood weight ratio of placing a root on the branch. The true root branch is indicated in red

**Fig. 7:**
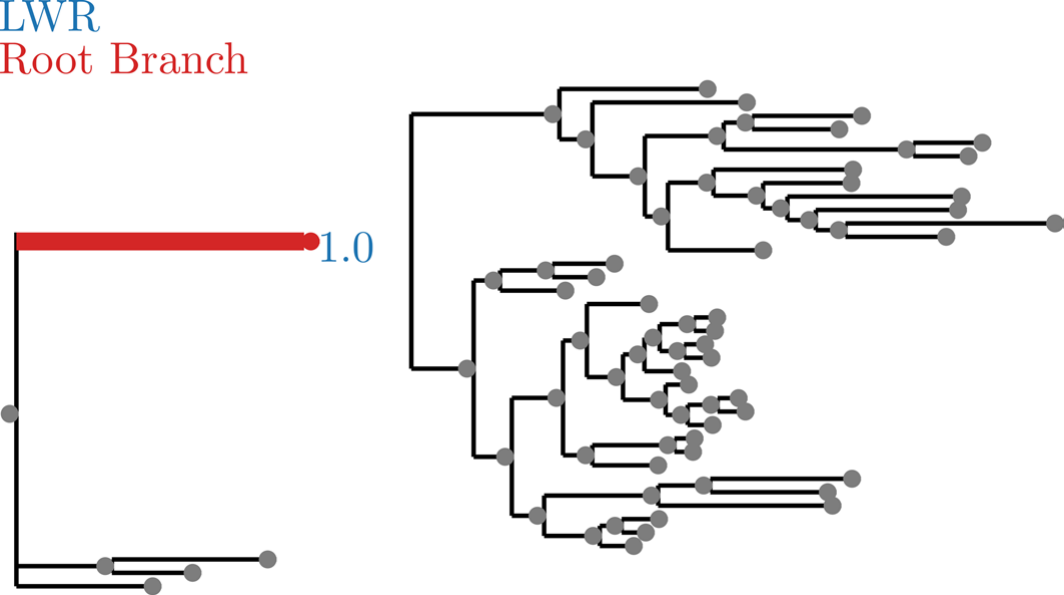
AngiospermsCDS12 dataset analyzed with an outgroup. LWR is the Likelihood weight ratio of placing a root on the branch. The true root branch is indicated in red

**Fig. 8:**
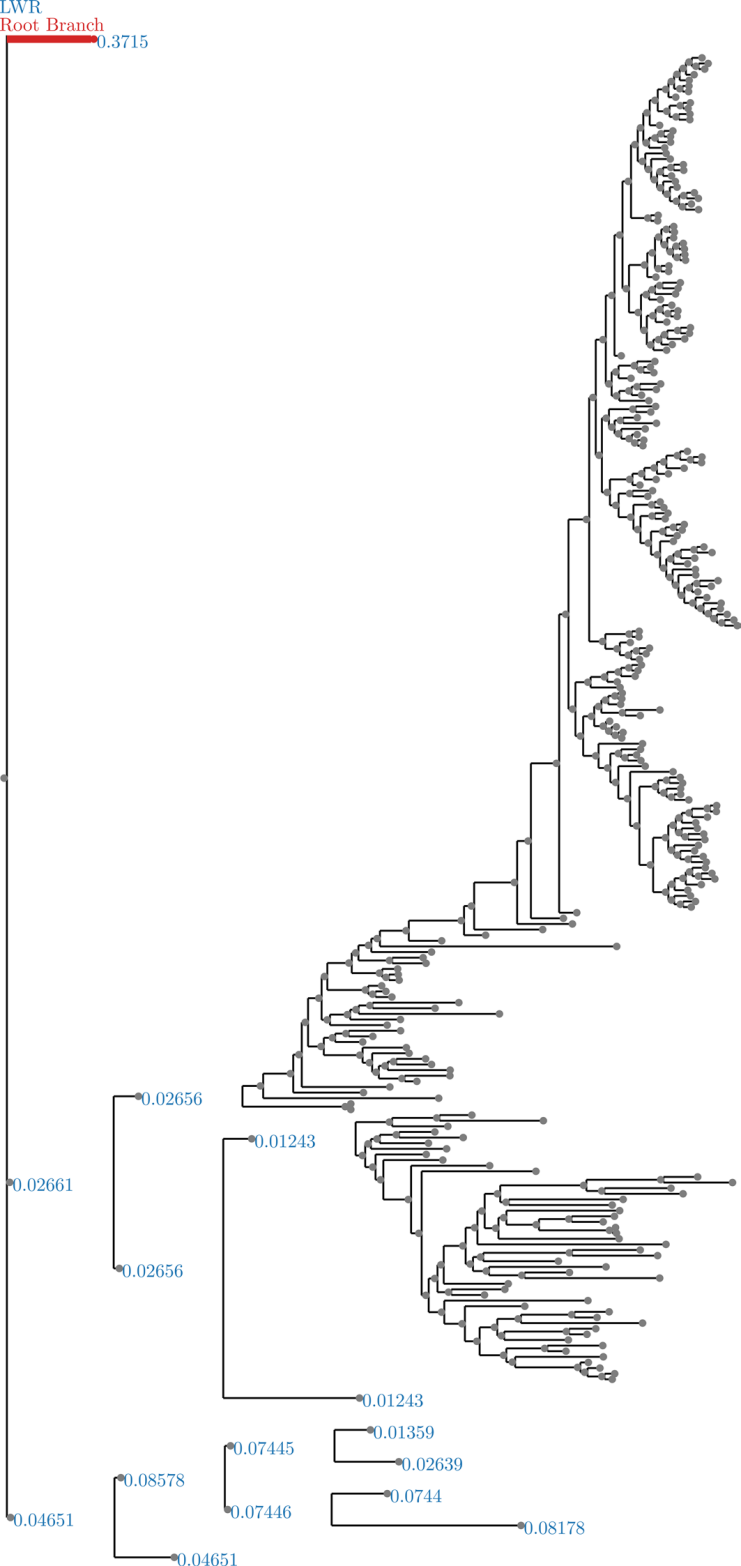
Grasses dataset analyzed without an outgroup. LWR is the Likelihood weight ratio of placing a root on the branch. The true root branch is indicated in red

**Fig. 9:**
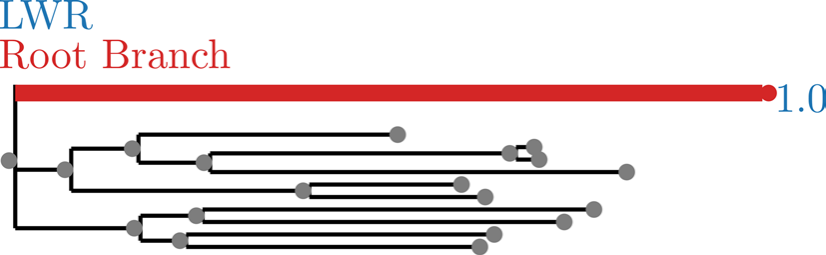
Beetles dataset analyzed without an outgroup. LWR is the Likelihood weight ratio of placing a root on the branch. The true root branch is indicated in red

**Fig. 10:**
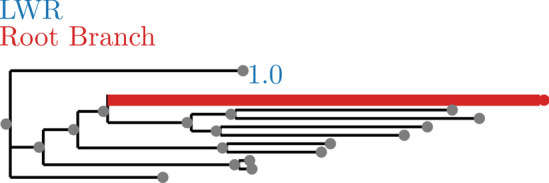
BeetlesHomogeneous dataset analyzed without an outgroup. LWR is the Likelihood weight ratio of placing a root on the branch. The true root branch is indicated in red

**Table 3:** Table of empirical datasets used for validation and results

Dataset	RD distance^abc^	IQ distance^a^	MAD distance
DS1	0.000	0.000	0.000
DS2	0.075	0.000	0.000
DS3	0.002	0.004	0.025
DS4	0.038	0.005	0.003
DS5	0.000	0.000	0.000
DS6	0.031	0.015	0.000
DS7	0.000^d^	0.000	0.000
DS8	0.158	0.000	0.000

RD distance and IQ distance are the average topological distances over 100 runs from the inferred root to the true root  normalized by the number of nodes (both tips and internal nodes). Similarly  for MAD the distance is also normalized by the number of nodes  but only 1 iteration was performed

^a^Averaged over 100 independent executions

^b^In early stop mode

^c^In search mode

^d^Results obtained using UNREST (without rate categories)

**Table 4:** Table of empirical datasets used for validation and results

Dataset	Search time^ab^	IQ-TREE time^ac^	Exhaustive time	MAD time
DS1	8.1m	48m	340m	0.00m
DS2	24m	114m	554m	0.00m
DS3	2.5m	1.5m	123m	0.02m
DS4	0.4m	0.4m	45m	0.00m
DS5	6.8m	25m	162m	0.00m
DS6	0.2m	1.5m	7m	0.00m
DS7	167m^d^	327m	441m	0.00m
DS8	4.8m	19.2m	81m	0.00m

Search and exhaustive times are for the respective modes of RootDigger

^a^Averaged over 100 independent executions

^b^In early stop mode

^c^In search mode

^d^Time obtained with UNREST+G4 (with rate categories)

### Effect of early stopping on result

**Fig. 11:**
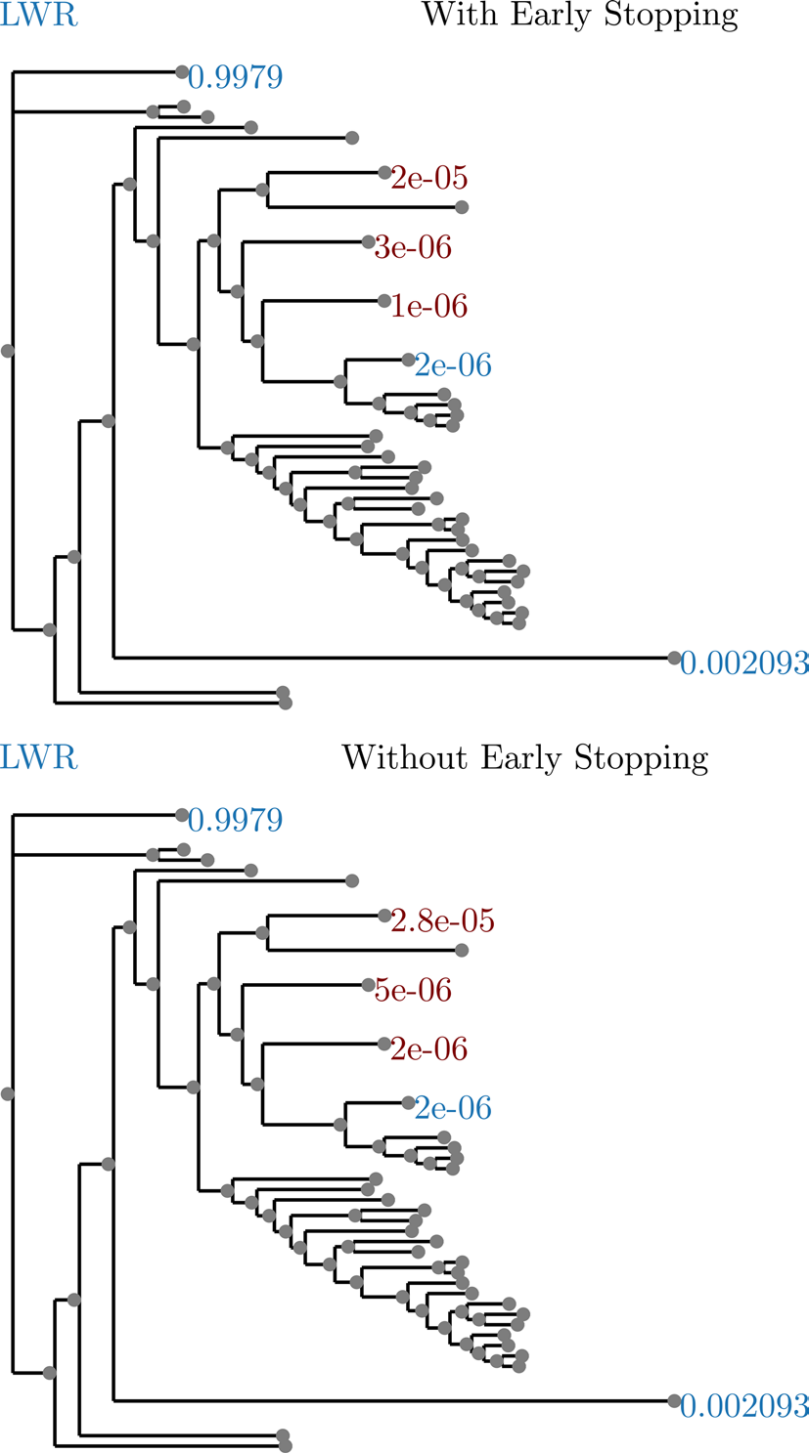
Effect of early stopping on results. Dataset is SpidersMitocondrial and has the largest observed difference of LWR between with and without early stopping

Finally, we investigated the effect of the early stopping criterion on the final LWR results. To do this, we ran RootDigger in exhaustive mode on all empirical datasets with early stopping enabled and disabled. For most runs, the results with and without early stopping showed no meaningful (difference in LWR less than 0.000001) difference. The dataset that showed the largest difference in LWR is shown in Fig. 11. In exchange, the runtime for this dataset with early stopping enabled is about 1.7 times faster.

Run time improvements for early stopping in search mode are less pronounced. We were not able to measure any significant difference in results or speed in search mode between early stopping enabled and disabled. We suspect that this is because the speed gain from early stopping in exhaustive mode is primarily due to it “skipping” low likelihood branches, which do not contribute significantly to the LWR.

### Parallel efficiency

**Fig. 12:**
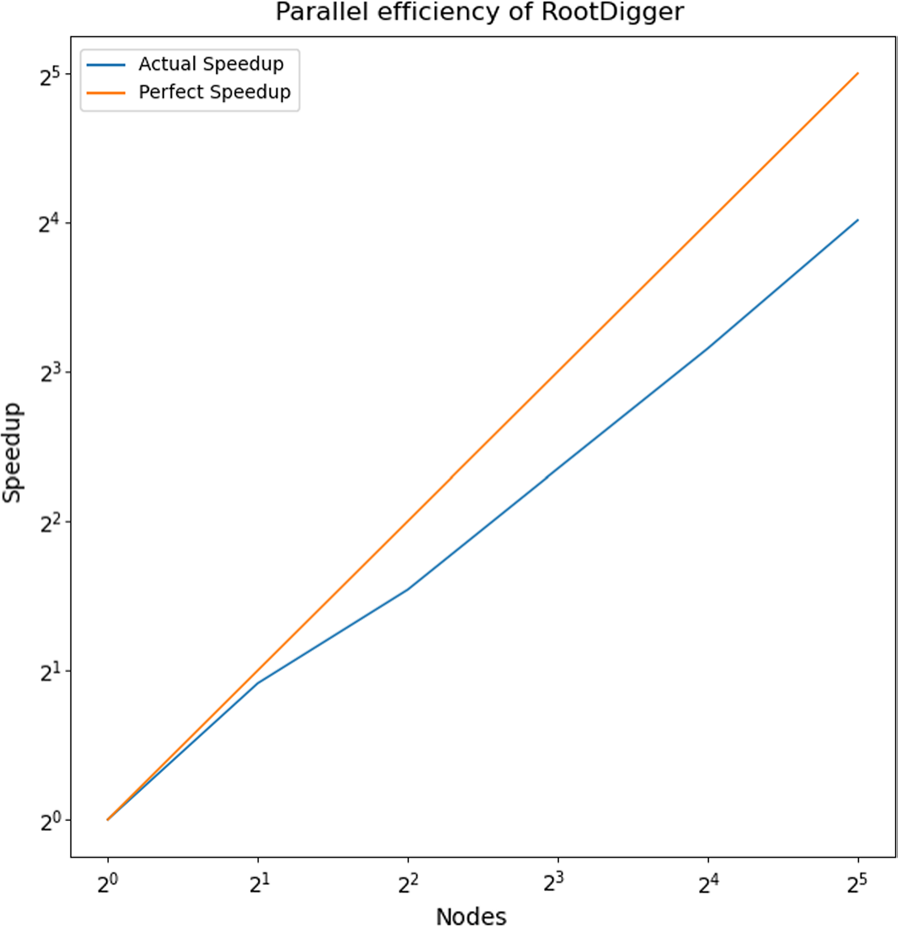
Plot depicting parallel efficiency, which is log nodes vs log speedup. Trials were run on 1, 2, 4, 8, 16, and 32 nodes with 16 threads per node using DS7 in exhaustive mode with early stopping turned off. The parallel efficiency ranges from 0.94 on 2 nodes to 0.50 on 32 nodes

Finally, we also evaluated the parallel efficiency of RootDigger. Figure 12 plots the speedup (how many times faster than 1 node) vs perfect efficiency for dataset DS7. We choose DS7 because it is one of the larger datasets at hand, and therefore is ideal for displaying the strengths and weaknesses of RootDigger’s parallelization strategy. Results were computed on a cluster, using MPI to communicate between nodes with RootDigger’s exhaustive mode. The parallel efficiency ranges from 0.94 on 2 nodes, to 0.50 on 32 nodes, each with 16 cores.

## Discussion

Compared to IQ-TREE, RootDigger performs competitively, as can be seen in both sides of Fig. 1. The results on simulations are mixed, with IQ-TREE performing slightly better in terms of root placement in all simulated scenarios. RootDigger is faster than IQ-TREE on all datasets we tested. When analysing empirical data, RootDigger also performed well, though not as well as IQ-TREE or MAD for most datasets, producing minimal errors in most cases. A notable exception is dataset DS3, for which RootDigger obtained a better result than either MAD or IQ-TREE. Examining the dataset with RootDigger’s exhaustive mode (see Fig. 8), we see that there is a number of branches with good support for a root placement. This suggests that there is conflicting signal as to the root location for this dataset, which naturally leads to confusion in generally reliable methods like MAD.

In general, exhaustive mode is more successful at identifying the correct root location (see Figs. 2, 3, 6, 7, 8, and 9). This is to be expected, since exhaustive mode performs a substantially more thorough search for the best root location. Nonetheless, this shows that RootDigger is successful not only at identifying the correct root location, but also at identifying any uncertainty or ambiguous signal for the dataset at hand.

Parallel Efficiency of RootDigger is acceptable, but could be further improved. Currently, it seems that losses in efficiency are largely due to the fact that different initial search locations require different amounts of time to complete. When this happens, some of the nodes finish early, and must wait for the remaining nodes to complete their computations. Due to this behavior, the parallel efficiency of RootDigger is dataset dependent. Fortunately, this behavior generally only manifests itself when each node has a small number of initial starting positions assigned to it. When this is the case, small variations in runtime are not given a chance to “average out” over many initial starting positions. In contrast, when a dataset is large with respect to the number of taxa, the number of initial starting positions increases and consequently the average time to complete computational work per node converges to an average amount. Nonetheless, RootDigger could benefit from a heuristic method to intelligently assign initial search locations to nodes.

## Conclusions

In Huelsenbeck [16], it was shown that the prior probability of a root placement on a sample tree did not have a strong signal when using a non-reversible model of character substitution. While performing our verification of RootDigger using empirical data, we found that this was often not the case. For example on the AngiospermsCDS12 dataset (see Fig. 6), we found a clear signal for the root placement, both with and without the outgroup.

Even in cases when the signal was not as strong, for example SpidersMitochondrial (see Fig. 4), there is a substantially stronger signal for root placement than the results in Huelsenbeck [16] would suggest we should obtain with this kind of analysis (which is to say, analysis using a non-reversible model). Those results in Huelsenbeck would suggest that we would essentially not be able to recover *any signal at all*. Instead, the signal appears to be moderately strong, at least most of the time. The one exception to this is the ficus dataset, which showed at least marginal support for the root on nearly all branches of the tree. We suspect that this is due to Huelsenbeck performing the analysis on a 4 taxon tree with the distantly related taxa frog, bird, mouse, and human. By only using 4 distantly related taxa, the rate matrix is less constrained by the data present, which may lead to over-fitting. In contrast, for “localized clades” we believe that we have shown that the methods presented here will typically produce a clear signal for the rooting of a tree, and when they do not we can identify such situations with the use of RootDigger’s exhaustive search mode.

Going forward with RootDigger, there are several developments that would be useful. One of these is the support for additional models. Currently, we only support the most complex model UNREST, but in the future it might be useful to support less complex models, such as the Lie group models described in Woodhams [24]. In particular, models with fewer parameters are generally regarded as being less prone to over-fitting, which might lead to a better assessment of the true root location.

In addition to more models, other data types could be supported, in particular amino acid (AA) data. In this work, we decided to not use AA data as it would increase the number of free parameters from 12 for DNA data to 380 for AA. Given this number, we suspect that it is too prone to over-fitting to be useful, but this has never been investigated.

Finally, there are a few parameters that are not part of the model that could be heuristically set in a less naïve way. These parameters include the number of initial candidate roots in the search mode and the number of roots to fully optimize during each step of the search mode. In this work these parameters were performing well on simulations, but better results could possibly be obtained via an adaptive strategy.

As mentioned in the discussion, the parallel efficiency of RootDigger could be improved using either of two techniques: Heuristically assigning initial search locations to nodes; or some dynamic scheduling of initial search locations to compute nodes. In the first technique, we attempt to estimate how long each root would take in relative terms, and then assign the initial search locations in such a way as to better balance the computational load. Traditionally, this can be quite difficult to do effectively, as the heuristic will often need to be finely tuned, which can cause degraded performance on atypical datasets. Alternatively, the initial search locations can be assigned dynamically. In this case, the initial search locations are passed out on demand, when a node has no computational work to conduct. From this point, it is not clear which method would perform better, and both should be investigated.

## Availability and requirements

Project name: RootDigger Project home page: https://www.github.com/computations/root_digger Operating system(s): Linux Programming language: C++ Other requirements: Bison/Flex, and optionally GNU Scientific library. License: MIT Any restrictions to use by non-academics: None.

## Data Availability

The datasets analysed during the current study are available in the root_digger_exp repository, https://github.com/computations/root_digger_exp.

## Abbreviations

AA: Amino acid
(L-)BFGS(-B): (Limited memory) Broyden–Fletcher–Goldfarb–Shanno (bounded)
DNA: Deoxyribonucleic acid
LWR: Likelihood weight ratio
MAD: Minimal ancestral deviation
MP: Message passing interface
MSA: Multiple species alignment
OpenMP: Open multi-processing

## Footnotes

1 Typically tree inference which uses AA data does not allow for using the fully unrestricted General Time Reversible (GTR) rate matrix, instead picking from one of several precomputed empirical substitution matrices. This substantially limits the number of free parameters, thereby reducing the risk for over fitting. In contrast, the fully unrestricted reversible AA rate matrix would have 380 free parameters. Therefore, we choose to limit RootDigger to DNA data because the equivalent model for AA data would have far too many parameters to reliably optimize.
